# Moving the fat: Emerging roles of rab GTPases in the regulation of lipid droplet contact sites

**DOI:** 10.1016/j.ceb.2025.102466

**Published:** 2025-04

**Authors:** Mariano Alonso-Bivou, Albert Pol, Harriet P. Lo

**Affiliations:** 1 Lipid Trafficking and Disease Group, Institut D'Investigacions Biomèdiques August Pi i Sunyer (IDIBAPS), 08036, Barcelona, Spain; 2 Department of Biomedical Sciences, Faculty of Medicine, Universitat de Barcelona, 08036, Barcelona, Spain; 3 Institució Catalana de Recerca i Estudis Avançats (ICREA), 08010, Barcelona, Spain; 4 Institute for Molecular Bioscience, The University of Queensland, Brisbane, Queensland, 4072, Australia

## Abstract

Lipid droplets (LDs) play crucial roles in lipid metabolism, energy homeostasis, and cellular stress. Throughout their lifecycle, LDs establish membrane contact sites (MCSs) with the endoplasmic reticulum, mitochondria, peroxisomes, endosomes, lysosomes, and phagosomes. LD MCSs are dynamically generated in response to metabolic or immune cues to ensure that LD lipids (and proteins) are timely delivered to optimize valuable substrates and avoid lipotoxicity. It is increasingly evident that many Rab GTPases are involved in LD dynamics. Here, we summarize our current understanding of how and when Rab proteins dynamically drive the generation of LD MCSs and regulate a variety of LD functions.

## Introduction

Lipid droplets (LDs) are highly dynamic organelles responsible for storage of fatty acids (FAs) and cholesterol in the form of neutral lipids, predominantly as triacylglycerol (TAG) and cholesterol esters [1]. LDs have a unique architecture consisting of a neutral lipid core surrounded by a phospholipid monolayer that accommodates a plethora of regulatory proteins. Originally regarded simply as sites of fat storage, LDs are now known to play crucial roles in diverse cellular processes ranging from lipid and energy homeostasis to innate immunity and inflammation [2,3].

The use of systematic molecular profiling approaches, capable of revealing and describing non-intuitive systems-level relationships, has uncovered an unexpected complexity in both function and regulation of LDs [4]. A proximity labeling strategy demonstrated that the LD-associated proteome encompasses a wide variety of proteins functionally grouped into metabolism, ubiquitin related, protein processing, cytoskeleton, autophagy, membrane organization, transcription, cell-signaling, and vesicular trafficking [5]. Particularly intriguing was the identification of 26 different Rab GTPases. Later, a comparative proteomic analysis of LDs purified from mice liver determined that the pool of LD-associated Rab GTPases is highly dynamic, with at least 22 Rab proteins upregulated on the LDs of cells activated by an inflammatory stimulus such as lipopolysaccharide (LPS) [6]. More recently, a multi-spectral organelle imaging approach determined that LDs form multi-organellar units with mitochondria, the endoplasmic reticulum (ER), and peroxisomes that are required for the metabolism of inflammatory lipid mediators [7]. Indeed, the function of LDs seems to be dependent on their physical interaction with other organelles via membrane contact sites (MCSs) and these processes are likely regulated by Rab GTPases. Such organelle–organelle communication could allow efficient trafficking of lipids to specific cell destinations, while preventing the lipotoxicity and oxidative stress resulting from lipid missorting [8].

The small GTPases of the Rab family have well established roles as molecular switches to control membrane trafficking and organelle–organelle communication in eukaryotic cells. The activity of these GTPases is regulated by their specific interactions with guanine nucleotide exchange factors (GEFs) and GTPase-activating proteins (GAPs) [9]. As many as 30 different Rab proteins have been linked to LDs through different proteomic studies. Importantly, the dysregulation of LD Rabs is associated with several genetic and metabolic diseases such as obesity, insulin resistance, and Warburg Micro syndrome (WARBM) [10]. Thus, it is timely to summarize our current understanding of the role of Rab proteins on LDs and revisit recent published data suggesting that LD-associated Rab proteins are involved in the formation, maintenance, and regulation of LD MCSs and hence emerge as key drivers of LD functioning.

### LD biogenesis, growth, and maturation: LD-ER contacts

The physical interaction between LDs and the ER is crucial for LD biogenesis and storage of FAs, cholesterol, and other lipids [11]. LDs arise from specialized ER subdomains acting as key sites of protein and lipid exchange and enriched in proteins required for LD formation [12]. FAs and cholesterol are esterified into neutral TAG and cholesterol esters by the sequential action of enzymes residing in the ER and gradually accrued into a lipid lens that phase separate within the ER bilayer to form a nascent LD. As additional lipid arrives and esterifies, the lens progressively grows into the cytosolic ER side to generate a spherical organelle encircled by a single monolayer of phospholipids containing the hydrophobic core of lipids [13,14]. Regulatory proteins are sequentially incorporated to the LD monolayer from the ER or, as in the case of perilipins and Rab proteins, from the cytosol [13, 14, 15∗∗, 16].

**Figure 1:**
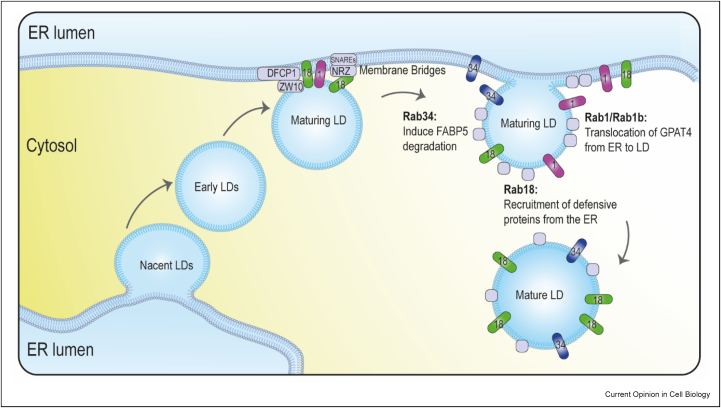
**LD-ER contacts allow the biogenesis and maturation of LD:** neutral lipids synthesized at the ER are accrued into a lipid lens that phase separate within the ER bilayer to form a nascent LD. The growing lens finally buds into the cytosolic ER side to generate a spherical organelle called early LD. Rab18 translocates from the ER to LDs and maintains the ER-LD membrane bridges for further LD growth. Membrane bridges are dependent on the ER-localized DFCP1 Rab18 effector or the NRZ-SNAREs complex that interact with the Rab18-ZW10 complex. These contacts may be necessary for the efficient transfer of defensive proteins from ER to LDs required for LD-dependent antimicrobial activity. Rab1 is implicated on late LD-ER membrane bridges and is essential for the targeting of GPAT4 from the ER to LDs. Rab1b was shown to be required for the redistribution of DGAT2 from the ER to the LD surface. Golgi resident Rab34 transits to the ER and then to the LD surface. From the LD surface, Rab34 regulates lipid accumulation and lipolysis by inducing the proteasomal degradation of the lipolytic enzyme FABP5. EE, early endosome; ER, endoplasmic reticulum; LD, lipid droplet.

Among LD Rabs, Rab18 has been the most extensively studied. Absent in nascent LDs, Rab18 is an ER/LD resident protein that participates in the formation of MCSs between the ER and mature LDs [17,18]. Although the function of Rab18 could be cell type–specific [19], 3T3-L1 preadipocytes without Rab18 have less mature LDs [19] and Rab18-deficient fibroblasts from WARBM patients accumulate abnormally large LDs [20]. Rab18 translocates from the ER to mature LDs by interaction with TRAPPII and COPI [21]. The ER-LD bridges mediated by Rab18 involve different complexes such as the ER-associated NAG-RINT1-ZW10 (NRZ) tethering complex and their associated SNAREs (Syntaxin18, Use1, BNIP1) [19] and the DFCP1-ZW10 complex [22] (Figure 1). Suggested by *in vitro* modeling, Rab18 MCSs could adjust the LD proteome in response to metabolic and immune cues [23]. These bridges may be necessary for the ER to LDs transfer of lipolytic enzymes or newly synthesized defensive proteins that, such as viperin, mediate the antimicrobial activity of LDs in response to interferon [6,24].

Another Rab protein functionally implicated on late LD-ER membrane bridges is Rab1, which is essential for LD growth. In a *Drosophila* model, Song et al. [15] described the early and late pathways of ER-to-LD protein targeting and identified Rab1 as essential for the targeting of GPAT4 and TAG synthesis [15,16]). More recently, in mammalian cells, Rab1b was shown to be required for the redistribution of DGAT2 from the ER to the LD surface, a process regulated by the GAP activity of TBC1D20 and involved in TAG synthesis and LD growth [25]. Interestingly, Rab18 and Rab1 are regulated by similar mechanisms; TRAPPII acts as a GEF for both Rabs and its depletion leads to defective LD metabolism [21]. Additionally, TBC1D20 has been confirmed *in vitro* as a Rab1 GAP with moderate activity on other LD-associated Rab proteins like Rab18 and Rab8a [26]. Mutations in this gene have also been associated with WARBM [27].

**Figure 2:**
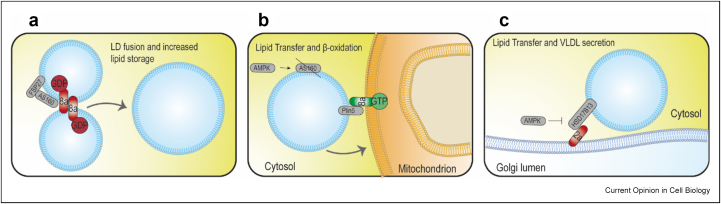
**Hormonal signaling regulates LD MCSs and metabolism through modulation of Rab proteins activity: (a)** LD-resident Fsp27 recruits active AS160 and Rab8a to LD–LD contact sites where AS160 converts Rab8a-GTP to Rab8a-GDP. Rab8a-GDP is then able to promote homotypic fusion of LDs to increase the storage capacity of lipids. **(b)** Under energy stress conditions, AMPK phosphorylates and inactivates AS160 that allows the recruitment of GTP-bound active Rab8a to mitochondrial membranes. GTP-bound active Rab8a anchored to mitochondrial membranes and PLIN5 on LDs membranes form a tethering complex that allows mitochondria–LD contacts. **(c)** Golgi-localized Rab2a mediates LD–Golgi interaction by binding LD-resident HSD17B13 and this may be regulated by AMPK signaling. EE, early endosome; ER, endoplasmic reticulum; LD, lipid droplet; MCSs, membrane contact sites.

The Rab-mediated regulation of the ER-LD MCSs determines the LD proteome and thus lipid metabolism upon different metabolic and stress situations (Figure 1). One example of this is Rab34, also recruited to LDs in late stages. The Golgi resident Rab34 transits via a COPI-mediated retrograde transport pathway to the ER and LDs to induce the proteasomal degradation of FABP5 and to inhibit lipolysis [28]. Furthermore, Rab2a has recently been implicated in the transfer of lipids from LDs to Golgi for VLDL secretion in hepatocytes [29]. Here, Golgi-localized Rab2a mediates LD-Golgi interaction by binding LD-resident 17-beta-hydroxysteroid dehydrogenase 13 (HSD17B13) and this may be regulated by AMPK signaling (Figure 2c). This interaction requires further research as the GAPs and GEFs for Rab2a remain to be identified.

### Regulation of lipid metabolism: LD–LD, LD-mitochondria, and LD-autophagosome contacts

Hormonal regulation of lipid and glucose metabolism in hepatic, heart, muscular and adipose tissues are of paramount importance in metabolic homeostasis. The canonical insulin signaling pathway, whereby activation of PI3K/Akt and subsequent translocation of GLUT4 leads to glucose uptake, has been studied extensively [30]. AS160 is a substrate for Akt and a known Rab GAP; its phosphorylation by Akt leads to Rab protein activation, GLUT4 translocation, and glucose uptake [31]. Intriguingly, Akt-phosphorylated AS160 also plays a role in LD dynamics. Fsp27 recruits AS160 and Rab8a to LD–LD contact sites [32], where AS160 can exert its GAP activity on Rab8a. In contrast with conventional pathways where the GTP-bound form of a Rab is the active form, AS160 converts Rab8a-GTP to Rab8a-GDP on the LD surface, which is then able to promote homotypic fusion of LDs to increase the lipid storage capacity (Figure 2a).

The interaction between LDs and mitochondria plays an important role during energy stress conditions such as starvation or exercise, where the release of FAs from LDs via lipolysis or lipophagy are used for energy production via β-oxidation in mitochondria [33]. Not surprisingly, LD–mitochondria contact sites are particularly abundant in metabolically demanding cells, including brown adipose tissue and muscle cells [12]. AMPK is a metabolic sensor that is activated in skeletal muscle under energy stress conditions and is known to increase lipid catabolism by increasing the uptake of FAs via CD36 [34] and mitochondrial oxidation via ACC2 [35]. AMPK alters the dynamics of LDs and, thus, their metabolism. Upon starvation, AMPK increases the mobility of LDs along detyrosinated microtubules, which enhances their interaction with mitochondria for FA oxidation and energy restoration [33]. Like AKT, active AMPK phosphorylates AS160 [36]. Phosphorylated and inactive AS160 allows the recruitment of GTP-bound active Rab8a to mitochondrial membranes where it acts together with Plin5 on LDs as a tethering complex that allows mitochondria-LD contacts [37]. Rab8a deficiency in skeletal muscle decreases FA oxidation during exercise and physical performance [37]. In this way, Rab8a arises as a crucial regulator of different membrane trafficking events by acting as a lipidic metabolism switch: GTP-bound Rab8a is anchored to mitochondria and acts as an LD receptor that leads to lipid transfer and β-oxidation, whereas GDP-bound Rab8a is anchored to LDs allowing LD–LD recognition and fusion, thus increasing lipid storage (Figure 2b).

Under severe starvation conditions, the late endocytic marker Rab7 mediates the interaction of LDs with lysosomes and multivesicular bodies during the process of lipophagy for utilization of LDs as an energy source [38]. After this, LD-associated Rab10 recruits LC3-positive autophagy membranes in complex with EHBP1-EHD2 to further promote the engulfment of LDs into autophagosomes [39].

### LDs and endosomes: LD-early endosome and LD-phagosome contacts

Of the large number of Rab proteins linked to LDs, those which are also associated with the endosomal system are particularly enriched on the LD surface, an intriguing aspect that thus far lacks a clear biological mechanism [40]. Martin et al. [18] showed for the first time that LDs are in close apposition with endosomal compartments tagged with Rab5, Rab7, and Rab11. Later, Liu et al. [41] showed Rab5 decorating the LD surface by immunogold labeling and EM in purified LDs. Importantly, they were able to demonstrate that the docking of early endosomes (EEs) to LDs occurred in a GTP-bound Rab5-dependent manner. This organellar association was long overlooked until recently, when EM demonstrated the formation of distinct MCSs between transferrin-HRP-labeled EE tubules and LDs, and even three-way contact sites with the ER [42]. Although the biological function of LD-EE MCSs remains elusive, it was recently hypothesized that Rab5 mediates the recruitment of early endosomal and or multivesicular body compartments to the LD surface before lysosome fusion and LD lysosome-dependent catabolism [43].

A widely accepted dogma is that microorganisms exploit LDs, using their stored lipids for growth. Indeed, LD accumulation has long been a hallmark of intracellular pathogens including bacteria, viruses, and parasites [44]. Of note, dengue virus infection relies on Rab18-mediated targeting of fatty acid synthase to replication sites for enhanced FA biosynthesis [45]. Rab18 has also been implicated in the trafficking of hepatitis C virus core protein to LDs [46].

This view has been challenged by the increasing evidence that mammalian LDs have co-evolved their own counter mechanisms against intracellular pathogens. LDs possess antibacterial activity, assembling immune defensive proteins and forming membrane contacts with bacteria-containing phagosomes [6]. This included upregulation of 22 Rab proteins in response to LPS stimulation. In addition, the activity of Rab7 is necessary for LD-phagosome contact in *Mycobacterium tuberculosis* infection [47]. The precise mechanisms of this novel role for LDs in innate immunity, mediated by Rab-dependent interaction of LDs and phagosomes, has yet to be fully explored and requires further research.

## Future perspectives

Developments in molecular profiling and imaging technology have greatly enhanced our understanding of LD dynamics and their interactions with other organelles. The role of Rab proteins as key players in LD-organelle communication (summarized in Table 1) to remodel the LD proteome in response to metabolic and immune stimuli has been an exciting advance in the field. While we have summarized the major LD processes controlled by Rab proteins, there remain many mechanistic questions regarding the defining features of Rab protein biology, and their dynamic activation and deactivation. How are specific Rab proteins activated to control the membrane–membrane interactions and trafficking processes required for LD function in response to specific metabolic or defensive needs of cells? Importantly, once the contacts are formed, what are the lipids, phospholipids, or proteins that are exchanged at each particular MCS?

**Table 1:** Summary of Rab proteins involved in LD–organelle contacts.

LD MCS	Rab	Function	Reference
**LD**–**ER**	**Rab18**	Recruited to the surface of LD via COPI-TRAPPII tethering complex to the ER.	[21]
	**Rab18**	Interaction with the ER NRZ/SNAREs complex to promote LD growth.	[19]
	**Rab18**	Interaction with the ER DFCP1 effector to promote LD growth.	[22]
	**Rab18**	LD-ER MCS are reconstituted *in vitro* and dependent on Rab18. These contacts are metabolic and immune sensitive.	[23]
	**Rab1**	Active Rab1 is required for the targeting of late cargoes to LDs, such as GPAT4.	[15]
	**Rab1b**	Active Rab1b is required for the DGAT2 ER to the LD surface redistribution and LD growth.	[25]
	**Rab34**	Translocates from ER to LDs where it promotes FABP5 degradation.	[28]
**LD–LD**	**Rab8a**	Rab8a-GDP on the LD surface promotes homotypic fusion of LDs and increasing storage capacity.	[32]
**LD**–**mito**	**Rab8a**	GTP-bound active Rab8a on mitochondrial membranes forms a tethering complex with PLIN5 on LDs membranes.	[37]
**LD**–**EE**	**Rab5**	Active Rab5 regulates transient interaction of LDs with EE.	[41]
**LD**–**Mtb phagosome**	**Rab7**	Controls interaction of LDs with Mtb-containing phagosomes.	[47]
**LD**–**autophagosome**	**Rab10**	Forms a complex with EHBP1-EHD2 for the autophagic engulfment of LDs.	[39]
**LD**–**MVB/Lys**	**Rab7**	Rab7 activation promotes trafficking of MVBs and lysosomes to LDs.	[38]
**LD**–**Golgi**	**Rab2a**	Golgi resident Rab2a forms a complex with LD-resident HSD17B13 to promote lipidation and secretion of VLDL.	[29]

EE, early endosome; ER, endoplasmic reticulum; LD, lipid droplet; MCS, membrane contact site; Mtb, *Mycobacterium tuberculosis;* MVBs, multivesicular bodies; VLDL, very-low-density lipoprotein.

In conclusion, far from moving only by vesicular trafficking or freely diffusing in the cytosol, organelle–organelle contacts emerge as a general regulatory mechanism for lipid metabolism and distribution. As the main lipid storage organelles in eukaryotic cells, LDs are strategically located/equipped to control these circuits. This is indeed an emerging and exciting field and further research is needed to characterize the implications of these dynamic relationships in cell’s metabolic fitness and disease pathogenesis.

## Author contributions

MAB contributed to conceptualization, writing–original draft, and visualization; AP contributed to conceptualization, writing–review and editing, supervision, project administration, funding acquisition; and HPL contributed to conceptualization and writing–original draft.

## Declaration of competing interest

The authors declare that they have no known competing financial interests or personal relationships that could have appeared to influence the work reported in this paper.

## Data Availability

No data was used for the research described in the article.
